# Acute liver injury linked to an adulterated weight-loss product: Integrated clinical, chemical, and toxicological evidence supporting a highly probable causality as assessed by updated RUCAM (2016)

**DOI:** 10.1016/j.toxrep.2026.102273

**Published:** 2026-05-10

**Authors:** Ferdinando Lucas Góis, Fernando Novais Júnior, Daniela Carneiro de Oliveira, Vinícius Santos Nunes, Raymundo Paraná Ferreira Filho, Genario Oliveira Santos Júnior, Ademir Evangelista do Vale

**Affiliations:** a Postgraduate Program in Pharmaceutical Assistance (PPGASFAR), Faculty of Pharmacy, Federal University of Bahia (UFBA), Salvador, Brazil; b Department of Gastroenterology, Professor Edgard Santos University Hospital Complex (HUPES), Federal University of Bahia (UFBA), Salvador, Brazil

**Keywords:** Hepatotoxicity, Herb-induced liver injury, Drug-induced liver injury, Adulterated products, Updated RUCAM, Translational pharmacovigilance

## Abstract

**Background:**

Acute liver injury associated with unregulated products marketed as dietary supplements is an emerging toxicological and public health concern, particularly due to the increasing prevalence of chemically adulterated formulations.

**Case presentation:**

A case of severe acute hepatocellular injury with marked aminotransferase elevation following short-term exposure to an Internet-purchased weight loss product is described. Clinical evaluation excluded alternative etiologies, including viral, autoimmune, and structural causes of liver injury.

**Methods:**

Causality was prospectively assessed using the updated RUCAM (2016). Chemical characterization was performed using gas chromatography–mass spectrometry (GC–MS) and inductively coupled plasma optical emission spectrometry (ICP–OES), complemented by in vitro toxicity testing (*Artemia salina*).

**Results:**

The Updated RUCAM assessment yielded a score indicating a highly probable causal relationship between product exposure and liver injury. Analytical profiling has identified multiple undeclared synthetic pharmacologically active substances at supratherapeutic concentrations, confirming product adulteration. The experimental assays demonstrated marked biological activity, supporting the toxicological relevance of these findings.

**Conclusions:**

This case highlights the significant hepatotoxic risk associated with chemically adulterated and unregulated weight-loss products. The integration of clinical assessment, advanced analytical chemistry, and experimental toxicology provides a robust translational framework for causal inference in suspected product-related liver injury, and underscores the need for strengthened pharmacovigilance systems targeting illicit or unregulated products.

## Highlights

• Adulterated weight-loss product associated with acute hepatocellular injury.
• GC–MS and ICP–OES identified undeclared synthetic drugs and labeling inconsistencies.
• Convergent clinical, analytical, and toxicological evidence supports causal attribution.
• Findings demonstrate a direct link between chemical adulteration and clinical outcomes.
• A multidisciplinary approach strengthens causal inference in product-related liver injury.

## Graphical Abstract

**Figure:**
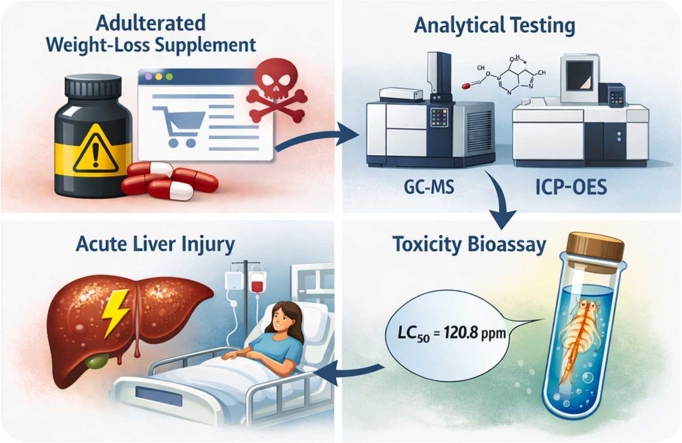

## 1 Introduction

Liver injury associated with herbal products represents an increasingly recognized clinical and toxicological challenge, driven by the global proliferation of unregulated and chemically adulterated formulations that differ fundamentally from regulated dietary supplements [1]. The updated Roussel Uclaf Causality Assessment Method (RUCAM, 2016) allows structured causality assessment at the level of the suspected product and, when sufficient data are available, may support the evaluation of individual components [2].

Increasing evidence indicates that hepatotoxicity attributed to herbal products is frequently driven not by intrinsic phytochemical toxicity but by intentional adulteration with undeclared pharmacologically active substances [3]. Such adulteration blurs the distinction between herb-induced liver injury (HILI) and drug-induced liver injury (DILI), posing a major challenge to clinical hepatology, toxicology, and pharmacovigilance.

Weight loss products are consistently among the most frequently implicated hepatotoxic agents, especially when acquired through informal markets or online platforms [4], [5]. Studies have repeatedly demonstrated mislabeling and adulteration with anorectic agents, antidepressants, laxatives, and centrally acting drugs [6], [7]. These hidden compounds can amplify hepatotoxic risk through pharmacodynamic interactions, metabolic competition, and oxidative stress–mediated hepatocellular injury [8], [9], [10]. Despite this growing recognition, regulatory surveillance remains limited, with irregular, unregistered, and illegal products often escaping notifications and systematic monitoring.

Most published reports on product-related hepatotoxicity rely primarily on clinical causality assessment without incorporating chemical characterization or experimental toxicity evaluation of suspected products [5], [9], [11], [12]. This gap weakens the causal inference and obscures mechanistic understanding, particularly for adulterated formulations.

Translational approaches that integrate clinical hepatology, analytical chemistry, and experimental toxicology are urgently required to improve the detection, characterization, and risk assessment of hepatotoxic products [13], [14].

By integrating structured causality assessment with advanced analytical and toxicological approaches, this study provides translational evidence that chemically adulterated products are clinically relevant contributors to drug-induced liver injury (DILI) [15], [16]. These findings have implications for hepatology practice, particularly in the differential diagnosis of acute liver injury (ALI), and underscore the need for strengthened pharmacovigilance and toxicovigilance frameworks targeting unregulated or illicit products.

In this context, we report a case of severe ALI temporally associated with the use of an Internet-purchased weight loss product. Subsequent analyses revealed multiple undeclared synthetic pharmacologically active substances, including compounds subject to regulatory control in different jurisdictions. The coexistence of serotonergic, dopaminergic, anorexigenic, anxiolytic, and laxative agents in a product marketed as “natural” raises significant toxicological concerns.

It highlights the diagnostic challenges posed by concealed polypharmacy in irregular products, supported by comprehensive characterization - including gas chromatography-mass spectrometry (GC–MS), inductively coupled plasma optical emission spectrometry (ICP-OES), and *in vitro* toxicity - to strengthen causal inference by updated RUCAM. The clinical course is described in the following section.

## 2 Case presentation

A 39-year-old female retail worker, self-identified as a mixed race, 152 cm tall, weighing 80 kg, with no known comorbidities and no regular medication use, presented with acute neurological and systemic symptoms during a recreational trial hiking in the Paraguaçu River region (Recôncavo of Bahia, Brazil).

**Fig. 1:**
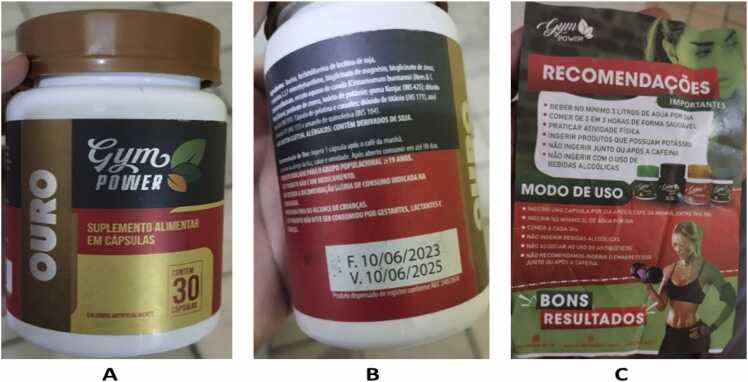
Secondary packaging (front and back labels) and informational leaflet of irregular product consumed by the patient. **Notes:** (A) Front label identifying the product as a “dietary supplement” and displaying marketing claims; (B) back-label listing declared plant-derived ingredients, vitamins, and minerals without quantitative composition or concentration data; (C) accompanying leaflet providing usage instructions and promotional claims. The labeling did not reveal the presence of synthetic pharmacologically active substances that were subsequently identified by chemical analyses. No valid batch numbers or sanitary registrations were identified in packaging.

Five days prior to symptom onset, she initiated daily ingestion of one capsule of an Internet-purchased weight-loss product marketed as “dietary supplement” (Fig. 1).

On May 18, 2024 (day 0), the patient developed vomiting during physical exertion, followed by sudden loss of consciousness, myoclonic jerks without sphincter release, dysarthria, and deviation of the labial commissure.

The patient received initial care at a primary healthcare unit (UPA) where the first laboratory evaluation revealed marked biochemical abnormalities. Laboratory tests revealed marked hepatocellular and muscle injuries, including aspartate aminotransferase (AST) 1.941 U/L, alanine aminotransferase (ALT) 1.186 U/L, and creatine phosphokinase (CPK) 10.975 U/L, and a transaminase-predominant pattern, consistent with hepatocellular injury (Table 1).

**Table 1:** Serial biochemical laboratory parameters during hospitalization and follow-up (May–September 2024).

Parameter* (Normal range)	**Day 1** **19 May 2024, UPA**	**Day 2** **20 May 2024, HUPES**	**Day 4** **22 May 2024, HUPES**	**Day 6** **24 May 2024, HUPES**	**Day 10 28 May 2024, HUPES**	**Follow-up 4 months, 19 Sept 2024, AMN**
**Creatinine** (0.8 – 1.5 mg/dL)	1.26	0.9	1.0	0.7	0.6	0.7
**Urea** (mg/dL)	58	24	33	17	10	12.3
**Albumin** (3.5 – 5.0 g/dL)	------		3.2	3.3		4.3
**Total Bilirubin** (0.2–1.3 mg/dL)	------	3.98	5.84	8.80	4.51	0.36
**AST** (15–46 U/L)	1.941	7.772	4.015	534	114	26.4
**ALT** (11–69 U/L)	1.186	5.094	4.852	2.292	632	20.7
**ALP** (38–126 UI/L)	43.8	74	106	73	-------	-------
**GGT** (12 – 58 U/L)		140	171	209	-------	29.3
**CPK** (Women: 33–211 U/L)	10.975	9.587	1.492	205	277	
**LDH** (120 – 250 U/L)	-	-	1019	322	-	-
**Platelets** (150–450 ×10^3^ µL)	171.000	97.000	80.000	90.000	218.000	418.000
**PT** (> 70%)	-----	52.8	19	-------	79	-
**INR** (≤1.2)	-----	1.6	4.0	-------	1.18	1.18

**Abbreviations:** UPA, Emergency Care Unit (Unidade de Pronto Atendimento); HUPES, Professor Edgard Santos University Hospital Complex, Federal University of Bahia (UFBA); AMN, Magalhães Neto Outpatient Clinic (Ambulatório Magalhães Neto); AST, aspartate aminotransferase; ALT, alanine aminotransferase; GGT, gamma-glutamyl transferase; ALP, alkaline phosphatase; CPK, creatine phosphokinase; LDH, lactate dehydrogenase; PT, prothrombin time; INR, international normalized ratio.

Approximately 24 h later (May 19, day 1), she sought emergency care in Salvador because of persistent diplopia, blurred vision, recent memory impairment, diffuse myalgia, lower limb weakness, and malaise. The patient was referred to Professor Edgard Santos University Hospital Complex (HUPES) for specialized evaluation.

On hospital admission (May 20, day 2), rapid progression of liver injury was documented, with peak AST of 7.772 U/L and ALT of 5.094 U/L. Laboratory findings revealed hyperbilirubinemia (total bilirubin 3.98 mg/dL), thrombocytopenia (97,000/mm³), prolonged prothrombin time (52.8%), and an INR of 1.6. The patient was initially admitted to the Infectious Diseases unit and subsequently transferred to the gastroenterology unit. Serial laboratory data revealed a pattern of severe hepatocellular injury with transient coagulopathy and progressive hyperbilirubinemia followed by gradual biochemical recovery.

Extensive diagnostic investigations have excluded infectious etiologies. Serological testing was negative for hepatitis A, B, and C viruses; HIV; HTLV; and syphilis. Cytomegalovirus IgM was non-reactive, with reactive IgG consistent with past exposure. Herpes simplex virus hepatitis was considered because of the severity of presentation, and empirical acyclovir was administered for five days; However, subsequent clinical improvement and lack of supporting laboratory findings rendered this diagnosis unlikely.

Autoimmune hepatitis was considered improbable given its abrupt onset, absence of hypergammaglobulinemia, and favorable evolution without immunosuppressive therapy. Abdominal imaging demonstrated diffuse hepatic steatosis without structural abnormalities or biliary obstruction, and cranial computed tomography (CT) revealed no acute intracranial abnormalities.

Supportive management included intravenous crystalloid fluids and oral vitamin K (10 mg/day for four days) to address coagulopathy. Progressive biochemical improvement was observed, with a marked decline in aminotransferase levels, normalization of coagulation parameters, and reduction in creatine phosphokinase levels.

By Day 10, substantial laboratory improvement was documented, and the patient was discharged for outpatient follow-up at the Magalhães Neto Outpatient Clinic (AMN), part of the HUPES. At four-month follow-up (September 2024), complete clinical and biochemical recovery was confirmed (Table 2).

**Table 2:** Timeline of clinical events and laboratory findings.

**Time point**	**Date**	**Event**
Days −5–0	13–17 May 2024	Daily ingestion of one capsule of an internet-purchased weight-loss product
Day 0	18 May 2024	Onset of vomiting, syncope, muscle jerks, dysarthria, and labial deviation during physical activity
Day 1	19 May 2024	Marked elevation of AST, ALT, and CPK detected
Day 2	20 May 2024	Hospital admission; peak hepatocellular injury with coagulopathy and thrombocytopenia
Days 4–6	22–24 May 2024	Progressive hyperbilirubinemia, peaking at 8.80 mg/dL
Day 10	28 May 2024	Significant biochemical improvement
Month 4	September 2024	Complete recovery

**Note:** AST = Aspartate aminotransferase; ALT = Alanine aminotransferase; CPK = Creatine phosphokinase.

The medication history revealed no regular prior drug use. The patient denied smoking and reported sporadic alcohol intake (approximately 88 g/month). The temporal relationship, hepatocellular pattern of injury, exclusion of alternative etiologies, and marked improvement following product withdrawal strongly supported the diagnosis of drug-induced liver injury (DILI).

Written informed consent was obtained from the patient prior to the collection and analysis of the suspected product, which was submitted for chemical and toxicological evaluation.

## 3 Results

### 3.1 Causality assessment

Causality was prospectively assessed using the updated RUCAM (2016) yielded a score of 9 (Table 3 and Supplementary Table S1), indicating a highly probable causal relationship between exposure to the implicated irregular product and observed liver injury.

**Table 3:** Causality between exposure to the implicated product and liver injury was assessed using the updated RUCAM. Each domain was scored according to established criteria.

**Domain**	**Score**	**Justification**
Time to onset	+ 2	Symptom onset occurred 5 days after product initiation, consistent with drug-induced liver injury (DILI)
Course after withdrawal	+ 3	Marked decrease (>50%) in ALT levels within 8 days after cessation
Risk factors	0	No established updated RUCAM risk factors (age <55 years; no significant alcohol consumption
Concomitant drugs	+1	No concomitant hepatotoxic drugs reported
Non-drug causes excluded	+2	Viral, autoimmune, metabolic, and structural causes excluded through comprehensive evaluation
Previous hepatotoxicity	+1	Known hepatotoxic potential of sibutramine and antidepressants
Re-exposure	0	Not performed
**Total score**	**9**	**Highly probable**

**Note:** Causality grading: ≤0 (excluded), 1–2 (unlikely), 3–5 (possible), 6–8 (probable), and ≥ 9 (highly probable).

This classification was supported by a compatible temporal relationship, clear hepatocellular pattern of injury, systematic exclusion of alternative etiologies (including viral, autoimmune, and structural liver diseases), absence of concomitant medication use, and consistent clinical improvement following the withdrawal of the suspected product.

Given the strong temporal association and highly updated RUCAM score, chemical characterization of the implicated product was performed to investigate potential adulteration and the presence of pharmacologically active xenobiotics that could mechanistically explain hepatic injury. Two capsules from the same commercially obtained product were subjected to chromatographic and elemental analysis.

### 3.2 Chemical characterization and toxicological assessment

#### 3.2.1 Gas Chromatography–Mass Spectrometry (GC–MS)

**Fig. 2:**
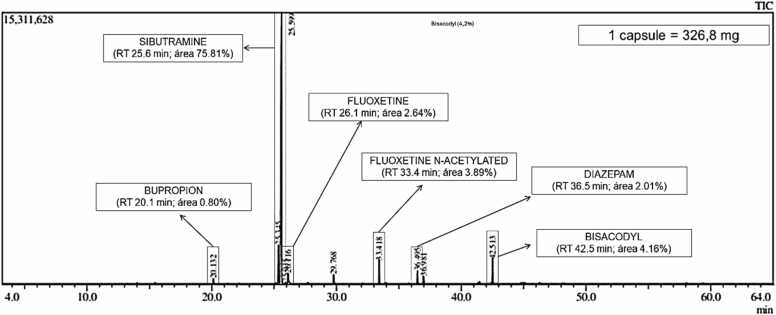
GC–MS chromatogram of the analyzed product indicating major undeclared synthetic pharmaceutical compounds.

Chromatographic profiling by GC–MS (Shimadzu QP2020; NIST14 library) revealed multiple undeclared pharmacologically active compounds. A representative chromatogram with peak assignments is presented in Fig. 2, and detailed chromatographic data are provided in the Supplementary Material (see Supplementary Figure S2A and Supplementary Table S2B).

The predominant peak was identified as sibutramine (75.8% of the total chromatographic area; approximately 247 mg/capsule). Additional detected compounds included fluoxetine (2.64%; ∼21 mg/capsule), N-acetylfluoxetine (3.89%; ∼30.9 mg/capsule), bisacodyl (4.16%; ∼14 mg/capsule), diazepam (2.01%; ∼6.5 mg/capsule), and bupropion (0.80%; ∼2.6 mg/capsule).

Compound identification was based on retention time matching and mass spectral fragmentation patterns, with similarity indices of > 90% relative to the reference library spectra. Representative mass spectral data for the major compound (sibutramine) are provided in Supplementary Figure S2C, which supports confident identification. It should be emphasized that the chromatographic analysis was semi-quantitative, and the compound levels were estimated based on the total mass of powder per capsule

The chromatographic profile demonstrated a predominance of pharmacologically active compounds, with only a minor fraction corresponding to non-active components, including triphenylphosphine (∼1.33%; ∼4.3 mg/capsule), which may be associated with manufacturing-related residues or formulation-related contamination.

#### 3.2.2 Inductively Coupled Plasma Optical Emission Spectrometry (ICP–OES)

Elemental quantification (Agilent 5800 ICP–OES) demonstrated discrepancies between the declared mineral composition and the experimental findings (Table 4), which integrated the ICP–OES and GC–MS results. Notably, the detection of non-declared elements and the absence of labeled constituents further support the inconsistencies in product formulation and quality control.

**Table 4:** Label-declared composition versus GC–MS and ICP–OES analytical findings in the evaluated product.

**Parameter**	**Declared composition (label)**	**GC–MS findings (RT, % area, mg/capsule); ICP–OES findings (ppm, and w/w)**
**Botanicals/** **Nutraceuticals**	Konjac (*Amorphophallus konjac*), Cinnamon (*Cinnamomum verum*), HMB (beta-hidroxi-beta-metilbutirato), Taurine, Phosphatidylserine, Tryptophan, Caffeine	Absence phytochemicals and nutraceuticals
**Synthetic drugs**	Undeclared	Sibutramine (RT 25.6 min; 75.81%; ∼247 mg); Fluoxetine N-acetylated (RT 33.4 min; 3.89%; 30.9 mg); Fluoxetine (RT 26.1 min; 2.64%; ∼21 mg); Bisacodyl (RT 42.5 min; 4.16%; ∼14 mg); Diazepam (RT 36.5 min; 2.01%; ∼6.5 mg); Bupropion (RT 20.1 min; 0.80%; ∼2.6 mg).
**Minerals**	Magnesium (Mg), Zinc (Zn), Potassium iodide (K), Chromium picolinate (Cr)	Zn: 16–17 ppm; K: 2.6–2.9% w/w; Mg: ∼0.17% w/w; Al: ∼300 ppm (undeclared); Pb, Cr < LOD

**Notes:** RT: retention time; GC–MS: gas chromatography–mass spectrometry; ICP–OES: inductively coupled plasma–optical emission spectrometry; LOD: limit of detection. Values are expressed as % area (GC–MS), mg/capsule, ppm, or w/w (%).

The analytical conditions included microwave-assisted digestion with concentrated nitric acid and hydrogen peroxide at 200 °C, followed by 50-fold dilution, ensuring efficient matrix decomposition and reliable elemental recovery. The detailed semiquantitative data are provided in the Supplementary Material (Supplementary Table S3).

#### 3.2.3 In vitro toxicity bioassay (*Artemia salina*)

The *Artemia salina* lethality assay demonstrated marked toxicity of the analyzed sample (120.8 ppm), providing complementary biological evidence consistent with the analytical findings [17]. These results support the presence of biologically active compounds detected by chemical characterization, and are aligned with the observed clinical features.

## 4 Discussion

### 4.1 Clinical presentation and causality assessment using the updated RUCAM

This report describes severe acute hepatocellular injury temporally associated with the ingestion of an irregular weight-loss product, which was subsequently demonstrated to be intentionally adulterated with multiple pharmacologically active synthetic substances [6], [12].

Although cases of liver injury are often categorized as herbal or dietary supplement (HDS)-related hepatotoxicity, this classification is not appropriate in the present case, as the investigated product does not meet the criteria for a regulated dietary supplement, but instead corresponds to an unregulated and chemically adulterated formulation.

The clinical presentation was characterized by marked elevations in aminotransferases, hyperbilirubinemia, coagulopathy, and thrombocytopenia, consistent with severe hepatocellular damage and paralleling patterns previously reported in chemically adulterated product–associated liver injuries [5], [8], [11]. Initial elevations in creatine phosphokinase levels were observed; however, the biochemical profile was inconsistent with that of isolated rhabdomyolysis.

An extensive etiological workup excluded viral hepatitis, HIV, HTLV, cytomegalovirus, and syphilis. Autoimmune hepatitis was considered unlikely based on its abrupt onset, the absence of hypergammaglobulinemia, and spontaneous recovery without immunosuppressive therapy.

In the present study, causality assessment yielded a score indicative of a highly probable association between product exposure and liver injury, underscoring the value of this structured framework for causality attribution not only at the product level, but also, when feasible, at the level of its individual components. Table 5 summarizes key study characteristics and highlights the predominance of ‘possible’ and ‘probable’ RUCAM classifications in the literature, in contrast to the present study.

**Table 5:** Selected studies contextualizing causality assessment in HILI/DILI using the updated RUCAM (2016) and complementary evidence frameworks.

**Study /Source**	**Product / Exposure**	**Evidence Framework**	**RUCAM Use**	**Typical Causality**	**Key Limitation**	**Key Contribution**
Danan & Teschke, 2016.	DILI/HILI (general)	Updated RUCAM (2016)	Yes (methodological)	---------	Data dependency	Standardization of causality assessment
Soares et al., 2021.	Herbal products (systematic review)	Algorithm comparison	Yes (mixed use)	Possible/ Probable	Incomplete datasets	Variability across causality tools
Bessone et al., 2022.	HDS (LATINDILI registry)	Observational registry	Yes (mixed use)	Possible/ Probable	Comorbidities, co-medication	Real-world complexity in HILI
de Assis et al., 2022.	Multiple HDS	Pooled case analysis	Variable	Possible/ Probable	Heterogeneous reports	Clinical severity and outcomes
Navarro et al., 2019.	HDS (USA cases)	Analytical + clinical	Not standardized	Variable	Mislabeling	Product inconsistency
LiverTox (NIH), Green tea.	*Camellia sinensis* extract	Evidence-based database	No	Probable / Known	Exposure variability	Established hepatotoxic risk
LiverTox (NIH), OxyELITE Pro®	Multi-ingredient supplement	Evidence-based database	No	High/ Definite	Adulteration	Strong causal association
Frenzel & Teschke, 2016.	Multiple herbs	Clinical compilation	Partial	Possible/ Probable	Case heterogeneity	Pattern recognition in HILI
Present study	Adulterated weight-loss product	Integrated: updated RUCAM + chemical authenticity + toxicological assays	Yes (updated 2016)	**Highly probable**	No hepatocyte-based in vitro assays; semi-quantitative GC–MS	High-confidence causal attribution supported by integrated clinical, chemical, and toxicological evidence

**Note:** RUCAM, Roussel Uclaf Causality Assessment Method; HILI, herb-induced liver injury; DILI, drug-induced liver injury; HDS, herbal and dietary supplements; GC–MS, gas chromatography–mass spectrometry.

In particular, incomplete information on product composition, uncertainty regarding the exact duration and timing of exposure, and the presence of potentially confounding factors, such as concomitant medications and pre-existing metabolic conditions, contributed to lower scores across key updated domains.

In the present case, the prospective application of the updated RUCAM (2016) enhances the reliability of causality assessment by improving temporal accuracy and minimizing recall bias.

Notably, even for well-established hepatotoxic products documented in LiverTox, causality attribution may remain variable, owing to similar constraints [12]. The present study illustrates that when analytical confirmation and detailed exposure data are available, the application of the updated RUCAM can support a highly probable causal relationship. Importantly, this high-confidence attribution is further strengthened by the convergence of chemical and biological evidence that supports the mechanistic plausibility of liver injury. This includes the detection of undeclared pharmacologically active compounds and their known hepatotoxicity [6], [8], [18].

Across different study designs, a predominance of “possible” or “probable” causality gradings is consistently observed, largely reflecting limitations in exposure characterization, data completeness, and product complexity [9], [11], [19].

In this context, the integration of clinical findings with temporal association supports a coherent toxicological framework linking exposure to hepatic injury, thereby justifying the need for detailed analytical characterization of the product.

### 4.2 Analytical chemical characterization

GC–MS analysis was used to identify the multiple undeclared synthetic drugs. Sibutramine was the predominant compound (∼75.8% chromatographic area; ∼247 mg/capsule), far exceeding the previously marketed therapeutic dose. Additional substances included fluoxetine, N-acetyl fluoxetine, bupropion, diazepam, and bisacodyl, consistent with intentional pharmaceutical adulteration, rather than incidental contamination.

This compositional pattern aligns with prior reports describing the adulteration of weight-loss products with centrally acting agents and psychotropic drugs, representing a recognized public health concern owing to their undeclared pharmacological activity and unpredictable toxicodynamic interactions [3], [7], [19]. From a mechanistic perspective, several of these compounds undergo extensive hepatic metabolism and have been associated with hepatotoxicity or intrinsic liver injury, supporting the biological plausibility of hepatocellular damage, particularly in the context of supra-therapeutic exposure and combined use [1], [6]

GC–MS also revealed minor compounds consistent with synthetic or manufacturing by-products, including triphenylphosphine oxide and long-chain aliphatic alcohols, suggesting poor manufacturing control and chemical mischaracterization. ICP–OES analysis revealed inconsistencies between the labeled and detected mineral compositions. Aluminum (∼300 ppm) was identified despite it not being declared on the product label, whereas chromium, which was listed on the label, was not detected. The lead levels were below the detection limit.

Although aluminum exposure is not typically associated with acute hepatotoxicity, its unexpected presence suggests possible contamination, manufacturing deficiencies, or poor raw-material quality [15], [16], [19]. Importantly, the elemental profile did not correspond to labeled claims, reinforcing the evidence of mislabeling and substantial quality control failures. As shown in Fig. 1, the product lacks proper labeling information, including batch traceability and disclosure of active compounds.

The integration of a structured clinical causality assessment with complementary analytical approaches provides compelling toxicological evidence that the investigated product is fraudulently formulated, chemically adulterated, and contains pharmacologically active substances.

### 4.3 Mechanistic plausibility

The five-day latency observed in this case is shorter than that typically described in non-adulterated herbal and dietary product–induced liver injury (HILI), suggesting a mechanism driven by synthetic pharmacological agents (DILI). Within the updated framework, this short latency is consistent with exposure to high-dose or rapidly acting compounds and contributes positively to the temporal relationship domain [20].

Sibutramine, a serotonin–norepinephrine reuptake inhibitor metabolized by CYP3A4, increases sympathetic activity and hepatic metabolic demand. The estimated sibutramine content (∼247 mg/capsule) substantially exceeds the previously marketed therapeutic doses (typically 10–15 mg/day), reinforcing dose-dependent hepatotoxic risk [18]. This supratherapeutic exposure supports the updated RUCAM domain related to drug-specific risks and strengthens the likelihood of a causal association.

Fluoxetine (a CYP2D6 inhibitor) and bupropion (a CYP2B6 substrate associated with reactive metabolite formation) may have contributed to the metabolic competition and enhanced oxidative burden [6], [8], [21], [22]. Although diazepam and bisacodyl are rarely hepatotoxic at therapeutic doses, their presence reflects uncontrolled polypharmacy that increases the likelihood of unpredictable toxicodynamic interactions.

Simultaneous exposure to serotonergic, dopaminergic, and noradrenergic agents at supratherapeutic concentrations supports the cumulative hepatotoxic potential mediated by oxidative stress, mitochondrial dysfunction, and cellular energy depletion [23].

The convergence of a high RUCAM score with results from a non-specific cytotoxicity screening assay (*Artemia salina*) provides complementary, mechanistic support for the observed association between exposure and liver injury, although it does not constitute direct evidence of hepatotoxicity or liver-specific effects.

### 4.4 Experimental toxicity evidence

Some botanical extracts may exhibit significant cytotoxicity in this model, including LC₅₀ values below; 50 ppm; however, authentic plant-derived preparations are more commonly reported to be weakly toxic or non-toxic [24], [25].

Therefore, although the contribution of plant constituents cannot be entirely excluded, the magnitude of toxicity observed here is more consistent with the presence of potent synthetic adulterants. This assay is widely used as a rapid screening tool for general cytotoxicity, but it does not replace clinical or in vivo studies for definitive assessment of hepatotoxic potential.

The integration of these biological findings with chemical characterization and clinical data strengthens causal inference, providing complementary evidence supporting the association between product exposure and liver injury.

In this context, the use of a simplified bioassay along with advanced analytical techniques reflects a translational toxicology approach applicable to the evaluation of chemically complex or adulterated products [24], [25], [26].

However, this study has some limitations. In vitro cytotoxicity assays were not performed in hepatocyte-derived models (e.g., HepG2), limiting the investigation of the cellular mechanisms of liver injury and potential synergistic effects among the detected compounds [27]. This limitation is particularly relevant in the context of multi-component and chemically adulterated products. In addition, the GC–MS analysis was semi-quantitative and did not allow precise determination of the absolute concentrations of the identified adulterants.

### 4.5 Regulatory and toxicovigilance implications

The intentional adulteration of unregulated or illicit products with pharmacologically active agents constitutes a form of food and health product fraud with clear toxicological and public health implications. Weight loss products distributed through informal or online markets are particularly prone to adulteration with anorectic, psychoactive, and laxative agents [28], [29].

The present case illustrates how the integration of structured clinical causality assessment, advanced chemical characterization, and experimental toxicological screening can substantially strengthen causal inferences in suspected product-related liver injury [3], [4], [30].

Such multidisciplinary approaches are especially relevant when exposure involves chemically mischaracterized or illegal products, for which conventional pharmacovigilance pathways are often insufficient [1], [3], [28].

From a public health perspective, these findings underscore the hepatotoxic risk associated with adulterated products, and highlight the importance of integrated pharmacovigilance and toxicovigilance systems. The convergence of structured causality assessment (RUCAM, 2016), advanced chemical characterization, and complementary experimental toxicity testing not only strengthens causal inference in suspected HILI/DILI cases, but also provides a model for translational investigation of product-related adverse events [12], [20], [31].

Beyond individual case management, this multidisciplinary approach generates critical evidence to guide clinical decision-making; inform regulatory policies; and contribute to global efforts in monitoring, reporting, and mitigating the public health impact of chemically mischaracterized or illicit dietary products [5], [12].

In particular, these findings underscore persistent gaps in regulatory oversight, including insufficient pre-market evaluation, lack of batch traceability, and inadequate post-market surveillance of “herbal products” [1], [30].

In a real-world context, the detection of undeclared synthetic pharmacologically active substances underscores the critical need for stricter quality control measures, systematic analytical screening, and enhanced enforcement actions targeting adulterated products.

While the updated RUCAM provides a structured clinical framework for causality assessment, integration with analytical confirmation of exposure and experimental toxicity data substantially strengthens causal inference. Strengthening collaboration among clinicians, toxicologists, and regulatory agencies is essential for improving data sharing, ensuring product accountability, and preventing recurrent exposure to hazardous formulations.

## 5 Conclusion

This report describes a case of severe acute hepatocellular injury associated with ingestion of a chemically adulterated weight-loss product in a university hospital setting. Analytical characterization confirmed the presence of multiple undeclared synthetic pharmacologically active substances at supratherapeutic concentrations, supported by experimental evidence of biological toxicity. A causality assessment using the updated RUCAM (2016) indicated a highly probable relationship between product exposure and liver injury.

These findings highlight the public health risks posed by unregulated and fraudulently formulated products, particularly those that lack manufacturing traceability and post-market surveillance. The integration of clinical assessment with advanced analytical techniques (GC–MS, ICP–OES) and experimental toxicity testing provides a robust framework for strengthening causal inference and supports improved pharmacovigilance and regulatory strategies.

## CRediT authorship contribution statement

**Ferdinando Lucas Góis**: Writing – review & editing, Writing – original draft, Visualization, Resources, Methodology, Investigation, Formal analysis, Data curation, Conceptualization. **Ademir Evangelista do Vale**: Visualization, Validation, Supervision, Resources, Project administration, Methodology, Investigation, Funding acquisition, Formal analysis, Conceptualization. **Genario Oliveira Santos Júnior**: Writing – original draft, Resources, Project administration, Investigation. **Raymundo Paraná Ferreira Filho**: Writing – original draft, Investigation. **Fernando Novais Júnior**: Investigation. **Daniela Carneiro de Oliveira**: Investigation. **Vinícius Santos Nunes**: Investigation.

## Consent for publication

Written informed consent was obtained from the patient. The document is available for review by the editor if requested.

## Declaration of Generative AI and AI-Assisted Technologies in the Writing Process

During the preparation of this work, the authors used ChatGPT (OpenAI) to assist with language refinement, grammatical revision, and improvement of scientific clarity. Following the use of this tool, the authors reviewed and edited the content as necessary and take full responsibility for the content of the published article.

## Funding

This study was supported by the Coordination for the Improvement of Higher Education Personnel (CAPES), Brazil (Finance Code 88887.920343/2023–00). F.L.G. received doctoral research funding from CAPES.

## Declaration of Competing Interest

The authors declare that they have no known competing financial interests or personal relationships that could have appeared to influence the work reported in this paper

## Data Availability

Data will be made available on request.
