# Supplementary material for: Acute liver injury linked to an adulterated weight-loss product: Integrated clinical, chemical, and toxicological evidence supporting a highly probable causality as assessed by updated RUCAM (2016)
Source: Toxicol Rep. 2026 May 10;16:102273. doi: 10.1016/j.toxrep.2026.102273 (PMC13195359; doi:10.1016/j.toxrep.2026.102273)
Supplement: Supplementary file 1 — Supplementary material [file mmc1.docx]

**ACUTE LIVER INJURY LINKED TO AN ADULTERATED WEIGHT-LOSS PRODUCT: INTEGRATED CLINICAL, CHEMICAL, AND TOXICOLOGICAL EVIDENCE SUPPORTING A HIGHLY PROBABLE CAUSALITY AS ASSESSED BY UPDATED RUCAM (2016)**

**Supplementary Material Overview**

This supplementary material provides detailed data supporting the clinical, analytical, and toxicological findings described in the main manuscript. It includes complete updated RUCAM scoring, comprehensive GC–MS analytical characterization, ICP–OES elemental analysis, in vitro toxicity assessment using the *Artemia salina* bioassay, and product documentation. These data complement the main text by enhancing transparency, reproducibility, and causal inference.

**S1. Updated RUCAM Detailed Scoring**

**Table S1. Updated RUCAM (2016) scoring for the GSL case (Gym Power - Gold).**

Causality between exposure to the implicated product and liver injury was assessed using the updated Roussel Uclaf Causality Assessment Method (RUCAM, 2016). Each domain was scored according to established criteria.

| **Domain** | **Score** | **Justification** |
| --- | --- | --- |
| Time to onset | +2 | Symptom onset occurred 5 days after product initiation, consistent with drug-induced liver injury (DILI) |
| Course after withdrawal | +3 | Marked decrease (>50%) in ALT levels within 8 days after cessation |
| Risk factors | 0 | \| No established RUCAM risk factors (age <55 years; no significant alcohol consumption \| \| --- \| |
| Concomitant drugs | +1 | No concomitant hepatotoxic drugs reported |
| Non-drug causes excluded | +2 | Viral, autoimmune, metabolic, and structural causes excluded through comprehensive evaluation |
| Previous hepatotoxicity | +1 | Known hepatotoxic potential of sibutramine and antidepressants |
| Re-exposure | 0 | Not performed |
| **Total score** | **9** | **Highly probable** |

**Note:** Causality grading: ≤0 (excluded), 1–2 (unlikely), 3–5 (possible), 6–8 (probable), ≥9 (highly probable).

**S2. GC–MS Analytical Characterization**

**Figure S2A. GC–MS chromatographic profile of the analyzed sample (Gym Power - Gold).**

The chromatogram shows a predominance of peaks corresponding to pharmacologically active compounds, with sibutramine as the major constituent. Additional active substances identified include antidepressants (e.g., fluoxetine, N-acetylfluoxetine, and bupropion), a benzodiazepine (diazepam), and other pharmacologically relevant agents. Minor peaks correspond to non-active components, including triphenylphosphine (~1.33%), possibly related to manufacturing residues or degradation processes.

The co-occurrence of multiple centrally acting agents, including catecholaminergic modulators such as sibutramine and bupropion, as well as serotonergic antidepressants (e.g., fluoxetine), may suggest a potential for pharmacodynamic interaction and increased hepatotoxic risk. In addition, fluoxetine is known to inhibit cytochrome P450 enzymes, which may further influence the metabolism of co-administered compounds and contribute to variability in toxic response.

**
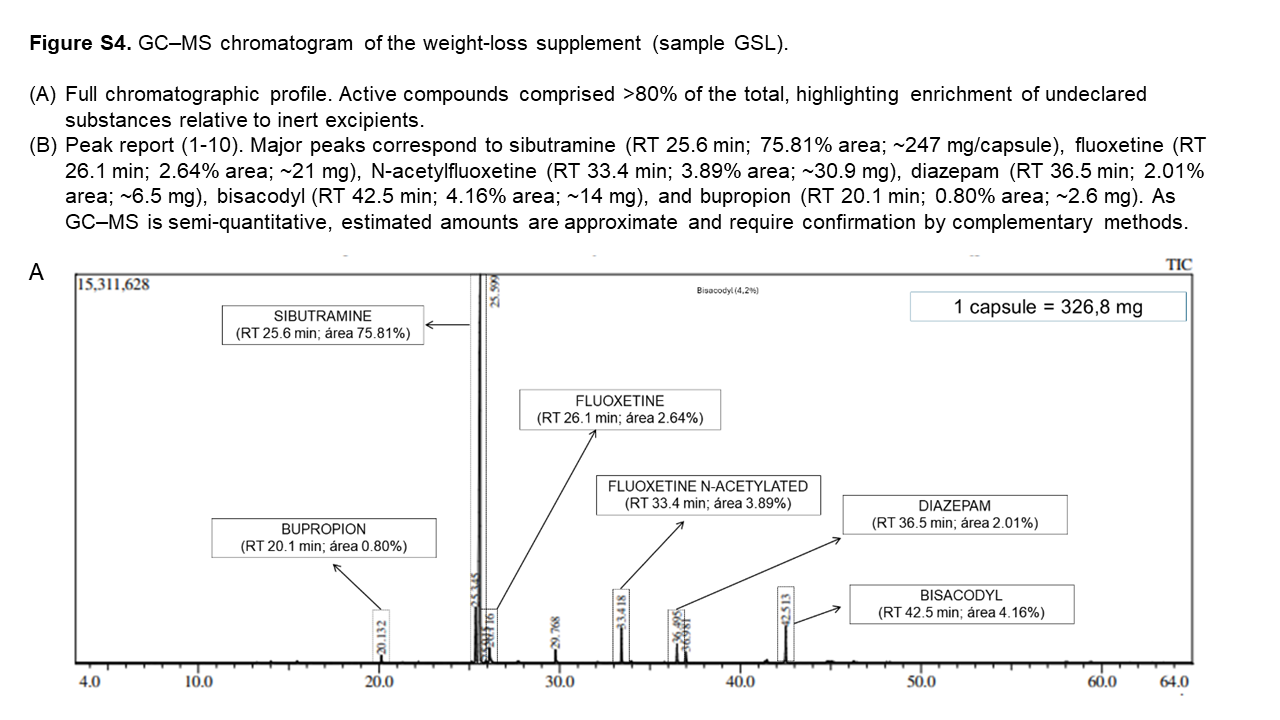
**

**Table S2B. Compounds identified in the analyzed sample by GC–MS.**

The table summarizes the compounds identified in the analyzed sample, including retention times, relative abundances, and estimated contents per capsule. The analytical profile demonstrates a predominance of pharmacologically active substances, with sibutramine representing the major component. The high relative abundance of active compounds, combined with the minimal presence of non-active components, supports the characterization of the product as an adulterated formulation containing undeclared synthetic drugs.

| **Compound** | **Retention time (min)** | **Relative abundance (%)** | **Estimated content (mg/capsule)*** |
| --- | --- | --- | --- |
| Sibutramine | 25.6 | 75.81 | ~247 |
| Fluoxetine | 26.1 | 2.64 | ~21 |
| N-acetylfluoxetine | 33.4 | 3.89 | ~30.9 |
| Diazepam | 36.5 | 2.01 | ~6.5 |
| Triphenylphosphine | 36.9 | 1.33 | ~ 4.3 |
| Bisacodyl | 42.5 | 4.16 | ~14 |
| Bupropion | 20.1 | 0.80 | ~2.6 |

*****Estimated values based on semi-quantitative analysis.

**Compound identification was based on:**

- spectral library matching (NIST14)
- retention time consistency
- diagnostic fragmentation patterns (>90% similarity)

**Figure S2C. Mass spectral identification of sibutramine.**

The mass spectrum of sibutramine (major chromatographic peak) demonstrated high concordance with reference library spectra, supporting confident compound identification. Diagnostic ions and fragmentation patterns were consistent with previously reported data.

(A) Ion fragmentation pattern showing characteristic ions

(B) Comparison with NIST14 reference spectrum demonstrating strong correspondence


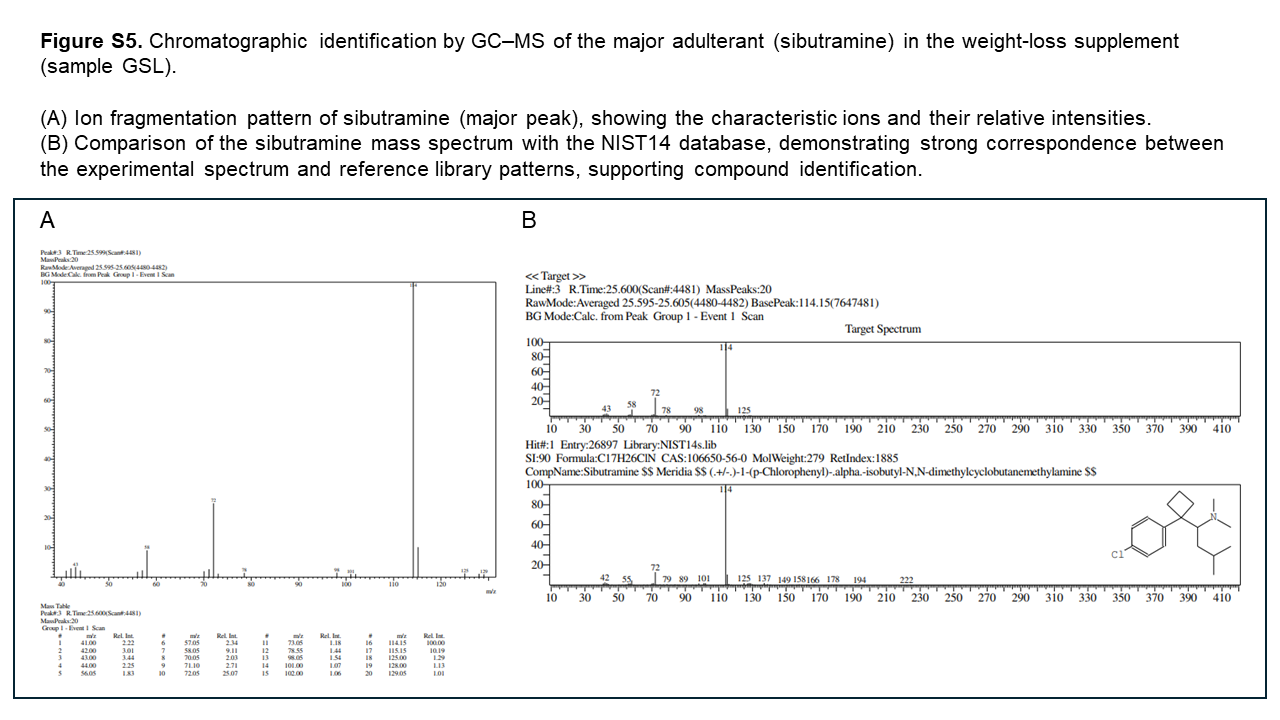


**S3. ICP–OES Elemental Analysis**

**Table S3. Elemental profile of the analyzed sample determined by ICP–OES.**

| **Element** | **Declared** | **Detected** | **Unit** |
| --- | --- | --- | --- |
| Zn | Yes | 16-17 | ppm |
| K | Yes | 2.6-2.9 | % (w/w) |
| Mg | Yes | ~0.17 | % (w/w) |
| Cr | Yes | <LOD | ------- |
| Al | No | ~300 | ppm |
| Pb | No | <LOD | ------- |

**Abbreviations:** ICP–OES, inductively coupled plasma optical emission spectrometry; LOD, limit of detection**.**

**Note:** Elemental analysis revealed discrepancies between labeled and detected composition, including the absence of declared chromium and the presence of undeclared aluminum, indicating inconsistency in product labeling.

**S4. In Vitro Toxicity Assessment (Artemia salina)**

**Table S4. Toxicity of the analyzed sample determined by the Artemia salina bioassay.**

| **Sample** | **LC_50_ (ppm)** | **Toxicological classification** |
| --- | --- | --- |
| GSL (Gym Power) | 120.78 | Moderately toxic |

**Toxicity classification followed Meyer and Clarkson criteria:**

- ≤100 ppm: highly toxic;
- 100–500 ppm: moderately toxic;
- 500–1000 ppm: low toxicity;
- 1000 ppm: non-toxic

**S5. Product Documentation (Gym Power)**

**Figure S5. Photographic documentation of the analyzed product (Gym Power; sample GSL) included:**

1. Front label with marketing claims
2. Back label listing declared ingredients without quantitative composition
3. Package insert with usage instructions


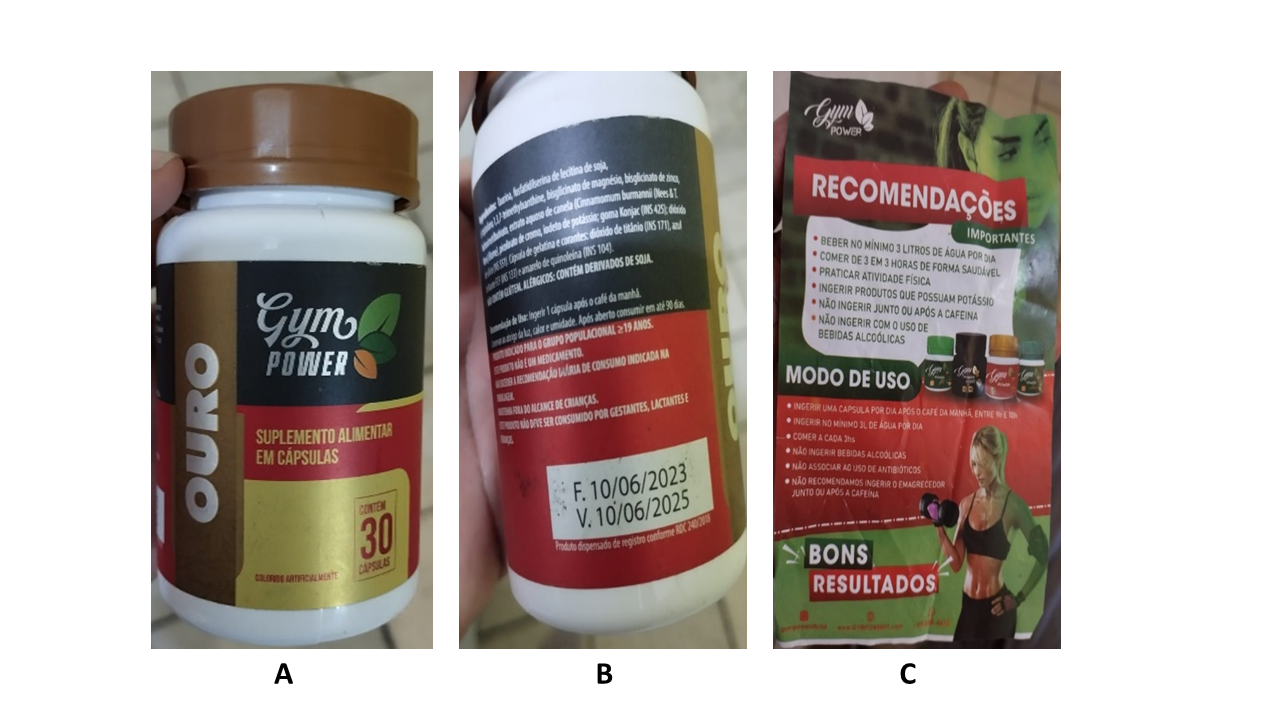
The product was marketed as a dietary supplement (herbal and dietary supplement, HDS); however, no valid batch number, manufacturer traceability, or sanitary registration was identified, indicating non-compliance with regulatory requirements. In addition, the presence of synthetic pharmacologically active substances detected by analytical methods was not disclosed on the label, further supporting the characterization of the product as irregular and potentially adulterated.

**S6. Methods Summary**

**S6.1 GC–MS Analysis**

GC–MS analyses were performed using a Shimadzu GCMS-QP2020 system operating in electron ionization (EI) mode (70 eV), with the injector maintained at 250 °C. Chromatographic separation was achieved using a capillary column (5% phenyl-methylpolysiloxane), with helium as carrier gas at a constant flow rate. The oven temperature program ranged from 60 °C to 280 °C. Samples were injected in split mode (split ratio as appropriate for matrix concentration). Data were acquired in full scan mode (m/z 37–660). Compound identification was based on comparison with the NIST14 spectral library and confirmation by fragmentation pattern analysis using Shimadzu GCMS Solution software.

**S6.2 ICP–OES Analysis**

Samples were digested using microwave-assisted acid digestion with nitric acid and hydrogen peroxide under controlled temperature and pressure conditions. Elemental quantification was performed using an Agilent 5800 ICP–OES system under standard plasma conditions. Multi-element calibration curves were constructed using certified reference standards, and quality control included reagent blanks and calibration verification standards. Analytical measurements were performed at selected emission wavelengths, with detection limits approximately 0.01 mg/L.

**S6.3** *Artemia salina* **Bioassay**

Cysts of *Artemia salina* were hatched in artificial saline solution (3.5% NaCl) under controlled conditions (25–28 °C, continuous aeration and light exposure). Nauplii were exposed for 24 hours to four concentrations of the sample (4.0, 2.0, 1.0, and 0.5 mg/mL). Samples were prepared using an appropriate solvent system to ensure solubility. Experiments were performed in triplicate, with 10 nauplii per replicate per concentration (total n = 120 per sample). Mortality was recorded after 24 hours, and LC₅₀ values were calculated using Probit regression analysis.
