# Supplementary material for: Acute liver injury linked to an adulterated weight-loss product: Integrated clinical, chemical, and toxicological evidence supporting a highly probable causality as assessed by updated RUCAM (2016)
Source: Toxicol Rep. 2026 May 10;16:102273. doi: 10.1016/j.toxrep.2026.102273 (PMC13195359; doi:10.1016/j.toxrep.2026.102273)
Supplement: Supplementary file 2 — Supplementary material [file mmc2.pdf]

Título do Estudo: Avaliação da atividade hepatotóxica *in vitro* deprodutos naturais utilizados por pacientes com suspeita de hepatotoxicidade em Centros de Referência em Hepatologia no Estado da Bahia.

Pesquisador Responsável: Genario Oliveira Santos Junior

TERMO DE CONSENTIMENTO LIVRE E ESCLARECIDO

O (A) Senhor (a) está sendo convidado (a) a participar de uma pesquisa. Por favor, leia este documento com bastante atenção antes de assiná-lo. Caso haja alguma palavra ou frase que o (a) senhor (a) não consiga entender, converse com o pesquisador responsável pelo estudo ou com um membro da equipe desta pesquisa para esclarecê-los.

A proposta deste termo de consentimento livre e esclarecido (TCLE) é explicar tudo sobre o estudo e solicitar a sua permissão para participar do mesmo.

O objetivo desta pesquisa é conhecer o potencial de dano hepático que os produtos naturais podem causar em pacientes atendidos Centros de Referência em tratamento de doenças hepáticas no Estado da Bahia. Este estudo pode possibilitar conhecer melhor quais são as espécies vegetais que são mais usadas e associadas ao dano hepático na população baiana , assim como , ajuda a desenvolver em laboratório experimentos que possam medir a toxicidade dos produtos naturais, ou seja , saber em qual dose podem comprometer a funcionalidade do fígado. A partir deste estudo podemos saber quais os produtos naturais podem levar a quadros mais graves como a insuficiência hepática aguda, e também conhecer as possibilidades de tratamento, além de poder gerar alertas de prevenção quanto ao uso de produtos naturais na sociedade.

Se o(a) Sr.(a) aceitar participar da pesquisa, os procedimentos envolvidos em sua participação são os seguintes: serão coletadas informações, através de um questionário, de modo complementar àqueles já colhidas durante a consulta médica sobre: a história clínica, exame físico, os dados laboratoriais, resultados de exames das sorologias virais, marcadores para auto anticorpos, diagnóstico de imagem e biópsia. Também será perguntado sobre uso do produto natural, onde foi local de coleta/aquisição do vegetal, o modo de preparo de uso, para que foi usado e por quanto tempo tomou, e o nome popular conhecido. De posse dessas informações, será solicitado ao senhor (a) que nos forneça, caso seja possível, uma amostra do produto natural para que possam ser feitos os estudos em laboratório.

Toda pesquisa com seres humanos envolve algum tipo de risco. No nosso estudo, os possíveis riscos ou desconfortos decorrentes da participação na pesquisa, estão relacionados aos procedimentos de coleta de informações e entrevista, o risco de constrangimento. Para isso, a entrevista da pesquisa será realizada em uma sala privativa, apenas com presença da equipe do Estudo, e para as perguntas que o participante não deseje responder, será respeitado o seu desejo. Também será utilizado a estratégia de codificação alfanumérico de modo que não seja possível identificar o participante.

O(a) Sr.(a) será acompanhado(a) a partir da data que aceitar participar do estudo pela equipe de saúde do seu Ambulatório ou hospital de origem treinado a realizar todos os procedimentos do estudo, e sua desistência e/ou término do estudo não implicará na sua assistência médica. Além disso, o(a) senhor(a) terá acesso e orientações quanto a todas as informações coletadas. A qualquer momento, se for de seu interesse, o(a) senhor(a) poderá ter acesso a todas as informações obtidas a seu respeito neste estudo, ou a respeito dos resultados gerais do estudo.

Contudo, esta pesquisa também pode trazer benefícios. Os possíveis benefícios resultantes

Rubrica do pesquisador | Rubrica do participante/responsável

Esse Termo é assinado em duas vias, sendo uma do(a) Sr.(a) e a outra para os pesquisadores.

Declaração de Consentimento

Concordo em participar do estudo intitulado: "Avaliação da atividade hepatotóxica *in vitro* de produtos naturais utilizados por pacientes com suspeita de hepatotoxicidade em Centros de Referência em Hepatologia no Estado da Bahia.".

Gilmara Santos Lucas<br>Nome do participante ou responsável<br><br>Gilmara Santos Lucas<br>Assinatura do participante ou responsável	Data: 18/07/2024

Eu, Geraldo Oliveira Junior, declaro cumprir as exigências contidas nos itens IV.3 e IV.4, da Resolução nº 466/2012 MS.

[signature]<br>Assinatura e carimbo do investigador	Data: 18/07/2024
